# Supplementary material for: cd1 Mutation in Drosophila Affects Phenoxazinone Synthase Catalytic Site and Impairs Long-Term Memory
Source: Int J Mol Sci. 2022 Oct 15;23(20):12356. doi: 10.3390/ijms232012356 (PMC9604555; doi:10.3390/ijms232012356)
Supplement: Supplementary file 1 [file ijms-23-12356-s001.zip › Supplementary Materials/Figure S1.pdf]

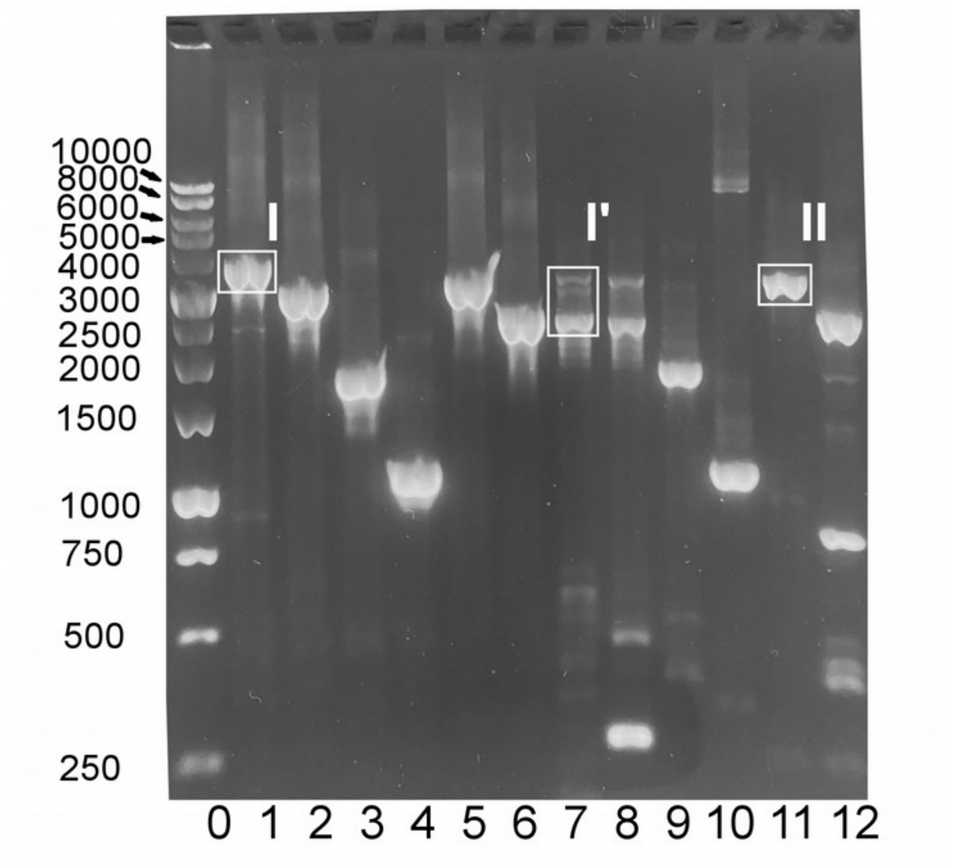

Figure S1. *cd* amplicons: PCR analysis.

0. 1 kb ladder.
Strains: 1 – 6: *CS*; 7 – 12: cd^{1}.

Primer pairs:
1: 1f – 7r (amplicon I for *CS*).
7: 8f – 7r (amplicon I for cd^{1}).
2, 8: 8f – 6r.
3, 9: 5f – 7r.
4, 10: 5f – 6r.
5, 11: 2f – 7r (amplicon II for cd^{1}).
6, 12: 2f – 6r.
