# Supplementary material for: cd1 Mutation in Drosophila Affects Phenoxazinone Synthase Catalytic Site and Impairs Long-Term Memory
Source: Int J Mol Sci. 2022 Oct 15;23(20):12356. doi: 10.3390/ijms232012356 (PMC9604555; doi:10.3390/ijms232012356)
Supplement: Supplementary file 1 [file ijms-23-12356-s001.zip › Supplementary Materials/Figure S2.pdf]

```
           1
reference  MTDETTPLTD AVPSGSGYVV LPPYQGPERV FPGGVSPRAR RNKMRQFQCC MGITFIAIVF TALCLALVFS
carinal    MTDETTPLTD AVPSGSGYVV LPPYQGPERV FPGGVSPRAR RNKMRQFQCC MGITFIAIVF TALCLALVFS

          71
reference  DSLGGADGGP SFFFVVNGSD SELAPNRPLP DEPAAEWALQ QAALGHHDGA QAVSAGIKAL GDREILEEGL
carinal    DSLGGADGGP SFFFVVNGSD SELAPNRPLP DEPAAEWALQ QAALGHHDGA QAVSAGIKAL GDREILEEGL

         141
reference  QPNEVNTPSF RHYRSLSTNP EARKLARRGY VENQATIDIA KRFNYTKQPG RSNIGWGPKI VLPDPTVLRL
carinal    QPNEVNTPSF RHYRSLSTNP EARKLARRGY VENQATIDIA KRFNYTKQPG RSNIGWGPKI VLPDPTVLRL

         211
reference  ECDFNARYRR STGVCNNKQH PRTYGASMVP YRRMVSPDYA DGIAAPRVSH HGRLPPARQV SLKIHRSSYE
carinal    ECDFNARYRR STGVCNNKQH PRTYGASMVP YRRMVSPDYA DGIAAPRVSH HGRLPPARQV SLKIHRSSYE

         281
reference  TDSNFTVMLA VFGQFMDHDI TATSLTTSQE GESIDCCVAA TREQHPECYP VDILPDDPYY KQYNISCMNF
carinal    TDSNFTVMLA VFGQFMDHDI TATSLTTSQE GESIDCCVAA TREQHPECYP VDILPDDPYY KQYNISCMNF

         351
reference  VRSAPAPTGR FGPRMQLNQA TAFIDASVVY GNLEQRQNQL RSFINGSLRM FVTDDGRQLL PISSNPADGC
carinal    VRSAPAPTGR FGPRMQLNQA TAFIDASVVY GNLEQRQNQL RSFINGSLRM FVTDDGRQLL PISSNPADGC

         421
reference  NRVQMTRLGK YCFESGDDRA NENLLLTSMH LLWARHHNYL ARQLQEQNPH WEDERLYQEA RKILGAQMAH
carinal    NRVQMTRLGK YCFESGDDRA NENLLLTSMH LLWARHHNYL ARQLQEQNPH WEDERLYQEA RKILGAQMAH

         491
reference  ITYNEFLPVL LGKNISEAKG LLPAKHNLNA PDTYDPEVDP SIANCFAAAA FRFAHTLLPG LFNISRDNST
carinal    ITYNEFLPVL LGKNISEAKG LLPAKHNLNA PDTYDPAILA LGGAWH

         561
reference  PEAIELHKML FNPFSLWAEH GIDHALMTAA NTPVMQVDRF FSLEVTQKLF EGTAEDRVPL CGLDLVSLNI
carinal

         631
reference  QRGRDHGIPS YPVFRRHCRL PTVDTWEEMS QAIDNATLDS IRQIYESPQD VDVYTGALSE PPLDGAIFGP
carinal

         701
reference  LLSCMVSDQF LRLKLGDSHW YERKMGPQKF TKAQLAEIYK TSLAAIICRN SDGITRVREH VMQRLRDGGN
carinal

         771
reference  PHVDCQDLEG FHFNFEPWSE KQQPQDLHSA GISRGSTSVR VMSKANHQAH NVTLHIDKGI
carinal
```

Figure S2. Alignment of the reference and *cd*[1] (amplicon III) PHS protein sequences.
