# Supplementary material for: cd1 Mutation in Drosophila Affects Phenoxazinone Synthase Catalytic Site and Impairs Long-Term Memory
Source: Int J Mol Sci. 2022 Oct 15;23(20):12356. doi: 10.3390/ijms232012356 (PMC9604555; doi:10.3390/ijms232012356)
Supplement: Supplementary file 1 [file ijms-23-12356-s001.zip › Supplementary Materials/Figure S3.pdf]

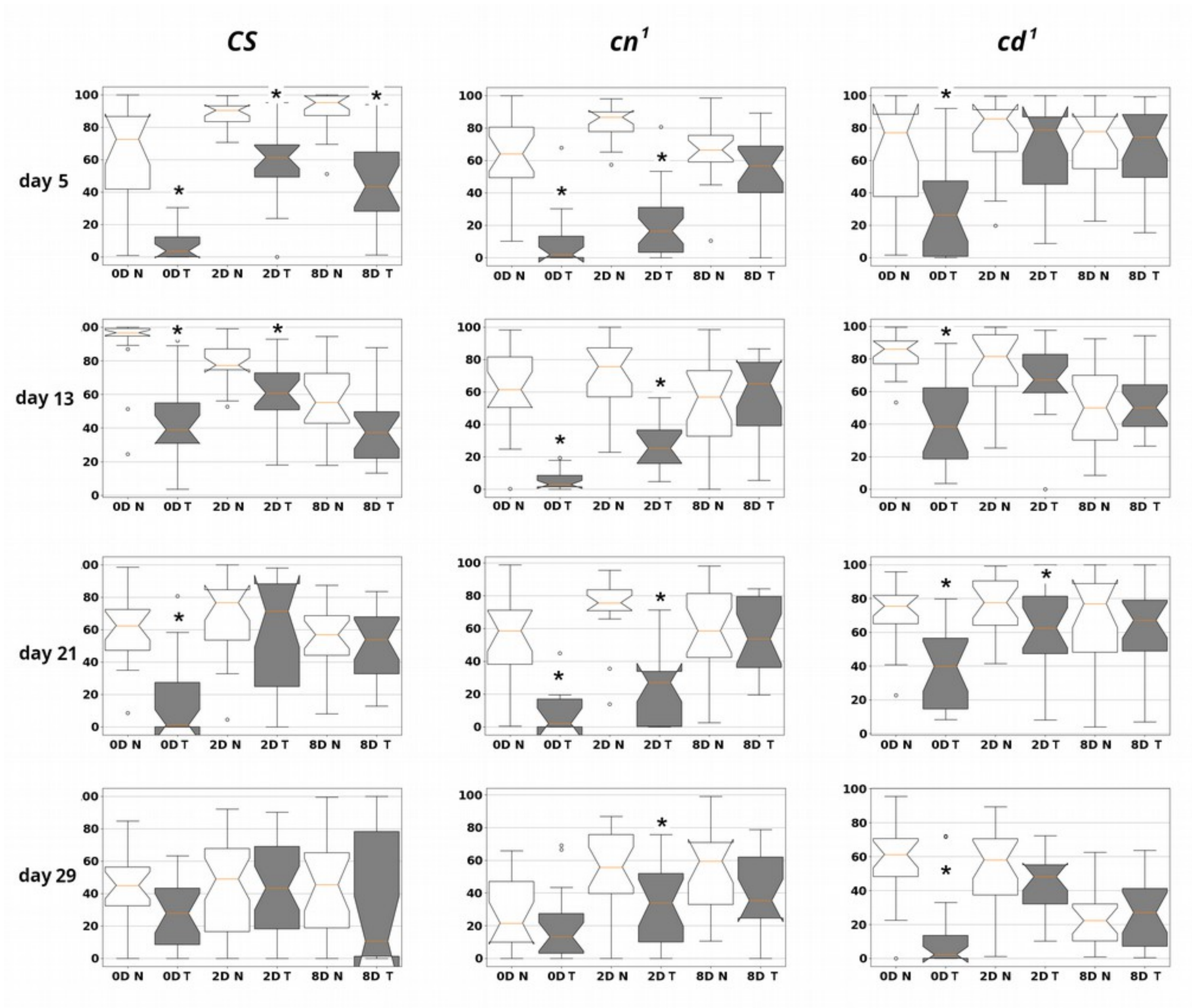

Figure S3. Age-dependent changes in courtship indices of kynurenine mutants.

N – naive flies, T – trained flies. X axis: days, Y axis: courtship indices (CI), %. Box and whisker plots are shown. 0D – immediately after training (learning), 2D – 2 days after training, 8D – 8 days after training. Median is shown by the orange line. Outliers are shown by dots. Statistical differences: * T from N of the same group (0D, 2D or 8D) (two-sided randomization test; $p < 0.05$, $n = 20$).
