# Supplementary material for: cd1 Mutation in Drosophila Affects Phenoxazinone Synthase Catalytic Site and Impairs Long-Term Memory
Source: Int J Mol Sci. 2022 Oct 15;23(20):12356. doi: 10.3390/ijms232012356 (PMC9604555; doi:10.3390/ijms232012356)
Supplement: Supplementary file 1 [file ijms-23-12356-s001.zip › Supplementary Materials/Figure S4.pdf]

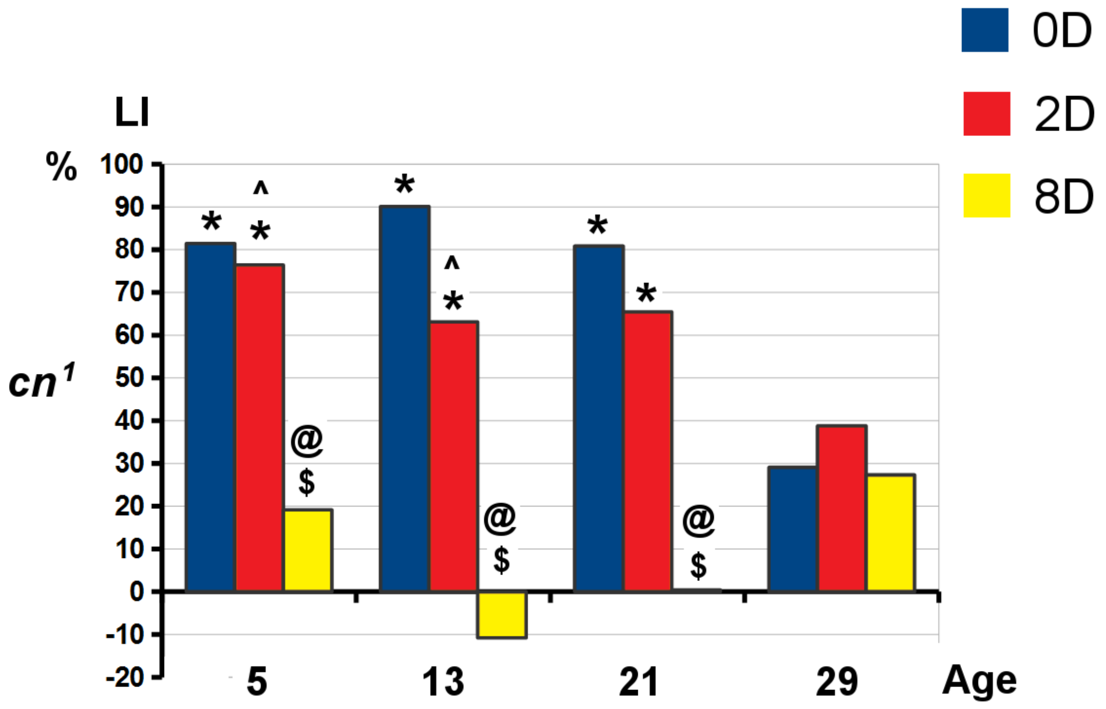

Figure S4. Learning and long-term memory abilities of *cn[1]* mutant.
X axis: the age where training was performed (days), Y axis: learning indices (LI), %. 0D – immediately after training (learning), 2D – 2 days after training, 8D – 8 days after training. Statistical differences: ^ from *cd[1]*; $ from 0D; @ from 2D; * from zero (two-sided randomization test; $p < 0.05$, $n = 20$).
