# Supplementary material for: cd1 Mutation in Drosophila Affects Phenoxazinone Synthase Catalytic Site and Impairs Long-Term Memory
Source: Int J Mol Sci. 2022 Oct 15;23(20):12356. doi: 10.3390/ijms232012356 (PMC9604555; doi:10.3390/ijms232012356)
Supplement: Supplementary file 1 [file ijms-23-12356-s001.zip › Supplementary Materials/Figure S5.pdf]

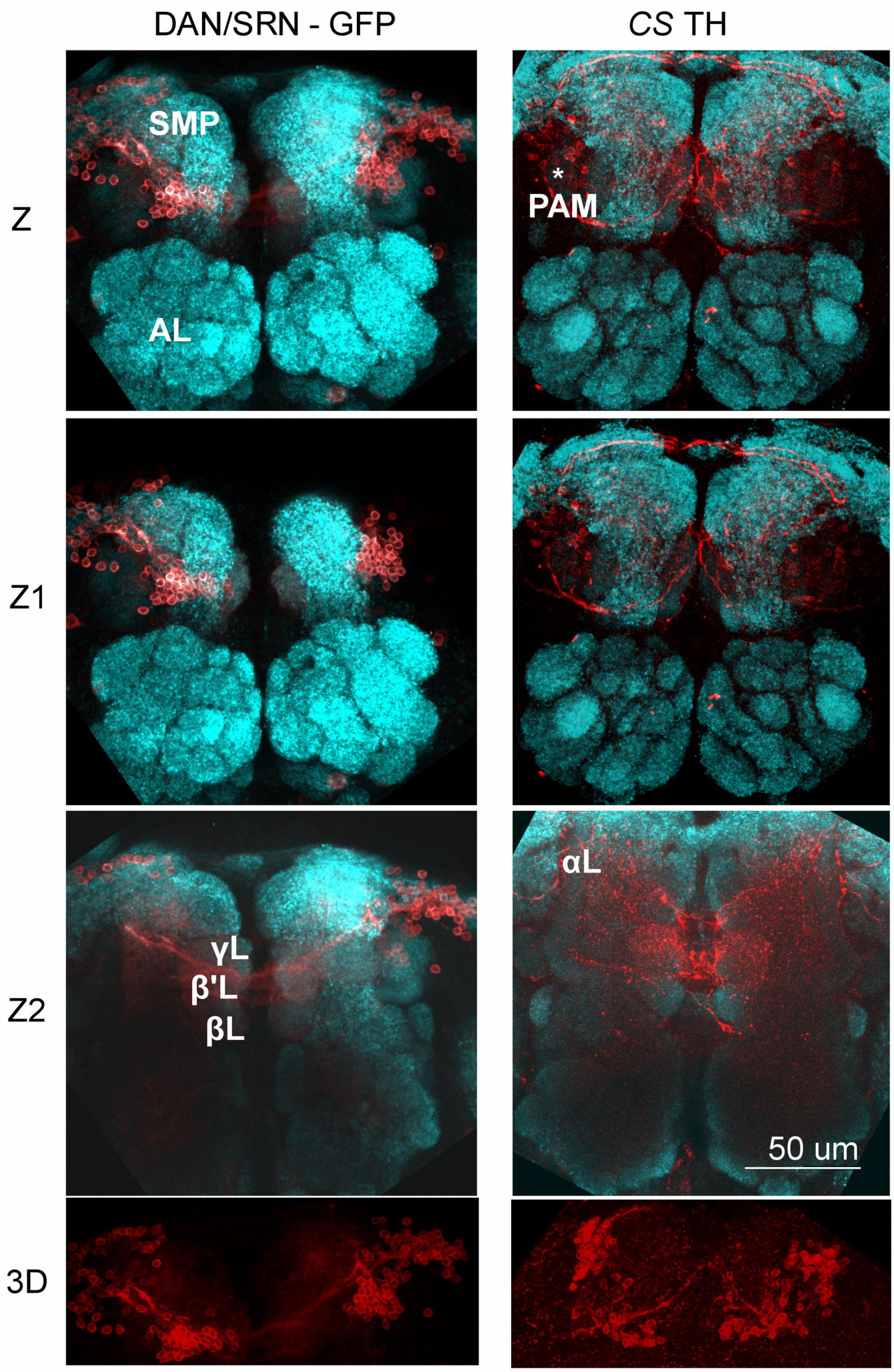

Figure S5. PAM neurons in the *Drosophila* brain.
DAN/SER – GFP: GFP signal in 7009 > 32186 strain (DAN ands SRN); TH – tyrosine hydroxylase. Z – Z-projection (Z1 + Z2). 3D – the spatial reconstruction of PAM clusters (*). Color scheme: cyan – CSP, red – GFP or TH. See also the list of abbreviations.
