# Supplementary material for: cd1 Mutation in Drosophila Affects Phenoxazinone Synthase Catalytic Site and Impairs Long-Term Memory
Source: Int J Mol Sci. 2022 Oct 15;23(20):12356. doi: 10.3390/ijms232012356 (PMC9604555; doi:10.3390/ijms232012356)
Supplement: Supplementary file 1 [file ijms-23-12356-s001.zip › Supplementary Materials/Text S1.pdf]

Text S1. Sequencing of *cd*: primers sequences and amplicons locations.

N	Forward (f)	Reverse (r)	Length	Location (bp)*
1	GACAGTTGCCTGCCTTGTTG	TGGTTAGCATGCTGCGGTTA	660	-156 – 503
2	AATTTGCCATCCCCCTCCATT	TCAGAGAGACCTGACGAGCC	766	416 – 1181
3	GTTCTGCCAGATCCTACGGT	TGCTCCAGATTGCCGTACAC	554	965 – 1518
4	GTTCTGCCAGATCCTACGGT	AGGTGATGTGTGCCATCTGA	503	1339 – 1841
5	TGCTGTGGGCTAGACATCAC	GTATGCCATGATCCCGACCC	564	1716 – 2279
6	GCAGAGGATAGGGTGCCTCT	TCCCTCACTCGGGTGATTCC	554	2204 – 2757
7	AGGTGACTCCCATTGGTACG	AAATCCACCCTTTCTCTGCGA	842	2566 – 3407
8	TCTTGGACGGTGGACAGTTG	GAAGGACGGCGTATTCACCT	983	-168 – 814
9	TTAAGGCACTGGGAGATCGG	TCTGCCGGATTCGAGGAAAT	874	744 – 1617
10	TCTGCCCGATGATCCCTACT	TGATCCCGACCCCTCTGAAT	909	1363 – 2271
11	TGATGCAAGTGGATCGCTTCT	GGTTACGTTGTGGGCTTGATG	806	2145 – 2950
12	TCTTGGACGGTGGACAGTTG^	CGTATCCGCCATTCTCGCTA	6234	-168 – 6065

N – the number of amplicon. ^ For N12, the forward primer is the same as for N8.
*Location (amplicon borders) is given relative to the point of *cd* start (3R:22694959, =1).

Polymerase chain reaction parameters
1. 95 ºC – 3 min: 1 cycle.
2. 95 ºC – 20'', 61 ºC – 30'', 68 ºC – X': 2 cycles.
3. 95 ºC – 20'', 59 ºC – 30'', 68 ºC – X': 2 cycles
4. 95 ºC – 20'', 57 ºC – 30'', 68 ºC – X': 2 cycles
5. 95 ºC – 20'', 55 ºC – 30'', 68 ºC – X': 38 cycles.
6. 68 ºC – 10 min: 1 cycle.
7. 4 ºC.
X = 1; 4 (for 1f – 7r, 8f – 8r, 2f – 7r); 11 (for 8f – 12r).
