# Supplementary material for: cd1 Mutation in Drosophila Affects Phenoxazinone Synthase Catalytic Site and Impairs Long-Term Memory
Source: Int J Mol Sci. 2022 Oct 15;23(20):12356. doi: 10.3390/ijms232012356 (PMC9604555; doi:10.3390/ijms232012356)
Supplement: Supplementary file 1 [file ijms-23-12356-s001.zip › Supplementary Materials/Text S3.pdf]

Text S3. Analysis of *cd* expression: primers sequences and amplicons location.

	Forward (f)	Reverse (r)	Length	Location (bp)*
rpl32	TATGCTAAGCTGTCGCACAAATGGC	GTTCTGCATGAGCAGGACCTCCA	195	365 – 559
cd	CACGTGGGAGGAGATGTCAC	CCACATCCTGCGGAGATTCA	99	416 – 1181

*Location (amplicon borders) is given relative to the point of *cd* start (3R:22694959, =1).

Real time polymerase chain reaction parameters
1. 95 ºC – 5': 1 cycle.
2. 95 ºC – 15", 62/58 ºC (*rpl32*/ *phs*) – 15", 72 ºC – 30", 83/78 ºC (*rpl32*/ *phs*) – 15" (detection): 40 cycles.
3. Melting curve analysis.
